# Abstract Concepts and Aging: An Embodied and Grounded Perspective

**DOI:** 10.3389/fpsyg.2017.00430

**Published:** 2017-03-22

**Authors:** Anna M. Borghi, Annalisa Setti

**Affiliations:** ^1^Institute of Cognitive Sciences and Technologies, Italian National Research Council (CNR)Rome, Italy; ^2^Department of Psychology, University of BolognaBologna, Italy; ^3^School of Applied Psychology, University College CorkCork, Ireland; ^4^Trinity College DublinDublin, Ireland

**Keywords:** abstract concepts, abstract words, words as tools, aging, elderly, embodied cognition, grounded cognition

How do we represent abstract concepts, as “justice” and “phantasy”? This issue has become hotly debated within embodied and grounded cognition views (for reviews: Pecher et al., 2011; Dove, 2016; Borghi et al., 2017). It is in fact unclear how such views can explain how we represent concepts that do not have single concrete referents and are rather detached from sensory experience (Barsalou, 2003; Binder, 2016).

In spite of the increasing interest for this issue, to date evidence on abstract concepts across the lifespan is limited. Assuming that the representation of abstract concepts changes from adulthood to older age, in this paper we discuss how a new embodied and grounded proposal, the Words As social Tools (WAT) view (Borghi and Binkofski, 2014), can explain how abstract concepts are represented by older individuals. More specifically we will advance hypotheses on abstract concepts in aging focusing on WAT, and reinterpret previous findings in light of it. We propose that WAT can account for existing findings and provide a suitable framework to test conceptual knowledge in older adults.

## Words As Social Tools (WAT)

According to WAT all concepts are grounded in perception, action and emotional systems; however, information conveyed through language in a social context is particularly crucial for abstract concepts while sensorimotor information is more crucial for concrete ones. Concrete concepts are typically acquired through sensorimotor interaction with the word referent. To acquire abstract concepts, instead, we benefit more of linguistic and social inputs, since their referents are not clearly defined objects/entities. The importance of language for abstract concepts is due to many reasons. First, labels can help, as a sort of glue, to keep together varied and heterogeneous experiences. Second, explanations offered by others are crucial in order to understand word meaning. Accordingly, abstract concepts are acquired later than concrete ones, they benefit more of linguistically conveyed information and are more influenced by the social context. Consistently, Age of Acquisition (AoA) and modality of acquisition, while being distinct properties of words, are both negatively correlated with concreteness and positively correlated with abstractness (Della Rosa et al., 2010). Third, inner language may help re-explaining to ourselves the meaning of abstract words, typically more complex than concrete ones (Borghi and Zarcone, 2016).

## Prediction

In order to advance predictions on abstract concepts in older adults derived from WAT, we refer to recent studies on healthy older, with no claim to be exhaustive.

Even if specific evidence on abstract concepts in healthy aging is lacking, there is evidence that linguistic abilities overall are preserved in elderly, although behavioral performance may be due to activation of different neural networks (Laumann Long and Shaw, 2000; Whiting et al., 2011; Shafto et al., 2012).

Based on the effect of the word AoA and of the word frequency, which are generally correlated (highly frequency words are typically early acquired words), one could predict higher difficulties in the mastering/use of abstract words in older adults, since these words are typically acquired later. When degradation of the semantic system occurs, frequent words and more imageable words are typically better preserved than less frequent words and less imageable words (Jefferies et al., 2009). However, it is to note that a reverse of the concreteness effect, i.e., the advantage in processing and recall of concrete over abstract concepts, has also been found (see Harciarek and Kertesz, 2011 for a review).

In absence of specific pathologies, since vocabulary is preserved with aging, abstract concepts' decline should be less marked than decline of concrete ones, since, compared to concrete concepts, they were mainly acquired through linguistically conveyed information and their representation relies more heavily on language. Hence, the concreteness effect should be reduced with aging (due to less steep decline of abstract concepts).

## Evidence

Evidence on concreteness effect in healthy aging is scarce.

A reduction of the concreteness effect with normal aging was found by Peters and Daum (2008) who investigated effects of aging on vivid remembering during recognition (recollection). They tested three groups of participants (mean ages 21, 42, and 61 years) in a deep encoding task: participants had to rate words for pleasantness. After an interval participants were required to respond “remember” whether they were certain that they had seen the word, and recollected associations, emotions etc. with it; and to respond “know” when they recognized the word but did not associate any further information with it. Recollection of concrete words declined progressively with age, while recollection of abstract words showed a decline only from the young to the middle age group, with no further decline between middle and older age, revealing the predicted reduction of the concreteness effect in the older group.

Shafto et al. (2012) with a lexical decision paradigm, instead, provided evidence for increased sensitivity to imageability, which is correlated with concreteness, in older adults more than in younger adults. Shafto et al. (2012) manipulated words imageability and phonology (measured by cohort competition) and recorded fMRI. They found preserved lexical abilities (capability to distinguish words from non-words) in older adults, and better performance with high imaginable words in both younger and older adults. Older participants manifested a higher sensitivity to imageability and a lower sensitivity to cohort competition, consistently with evidence showing a reduction of the phonological abilities after the mid 70's.

In a recent fMRI study, Roxbury et al. (2016) also conducted a lexical decision task, in which younger outperformed older adults (mean age 71) in response times and accuracy, and the concreteness effect was preserved with aging. However, the neural underpinnings differed for young and old adults, consistently with the hypothesis that compensatory mechanisms operate in elderly. Interestingly, activity of the left Inferior Frontal Gyrus (IFG), typically associated with phonological processes, increased for abstract words compared with concrete words, only for older adults. Results also showed an increased activity for abstract words in older compared with younger adults in the Left Fusiform Gyrus, an area associated to the retrieval of visual attributes (Binder et al., 2009) (differences were also found in the Angular Gyrus). It is to note that abstract concepts are characterized by visual attributes, while concrete concepts are characterized by different sensory modalities, particularly touch and somatosensation (Connell and Lynott, 2012).

One possibility is that the discrepancies are due to differences in the tasks: the concreteness effect seems to be reduced with age in recollection, but preserved in lexical decision. The difference might be due to the different level of processing the two tasks imply (superficial vs. deep), or to the emphasis placed on speed of processing, which is higher in the lexical decision task. While older adults have well preserved vocabulary, when they need to activate it fast they are disadvantaged due to their general cognitive slowness. However, they may be able to compensate better for words more defined in linguistic form than in perceptual form when the task is not based on speed.

Indeed, during the most superficial auditory lexical decision task (Roxbury et al., 2016) the concreteness effect was preserved at the behavioral level, but the neural underpinnings of the phenomenon differed between the older and the younger group. Specifically, the recruitment of left IFG in older adults for abstract words and pseudo words indicates that some kind of phonological processing takes place. This, according to WAT, could be associated with covert verbalization of the meaning. It is worth noting that phonological processing declines with aging (Burke and Shafto, 2004), however, older adults continue to rely on inner speech (Alderson-Day and Fernyhough, 2015).

As predicted by WAT, the activation of left IFG could reflect the activation of compensatory strategies, which involve linguistic conveyed and linguistic form information, both crucial for abstract concepts representation. The activation of the left IFG would thus play a compensatory role in particular for later acquired abstract concepts. Notice that, according to WAT, abstract concepts do not activate only linguistic information but are also grounded in perception, in particular in the visual modality. In spite of the decline of vision with age, recent evidence has shown that the importance of visual features compared to other sensory modalities is higher for older then for younger adults (Maguinness et al., 2013; Costello and Bloesch, 2017). This activation of the visual modality could be associated with the activation of the Left Fusiform Gyrus (Roxbury et al., 2016).

In sum: according to WAT abstract concepts are grounded in sensorimotor and in linguistic/social experience. Consistently, the loss of abstract words, which are typically later acquired and less frequent than concrete ones, would be compensated reactivating phonological/linguistic and visual features. Based on WAT we propose that abstract and concrete concepts should have a different trajectory of decline. When, with older age, the decline starts to occur, concrete concepts, relying more on sensorimotor and episodic knowledge, should decline faster, while abstract concepts should decline slower since they rely also on linguistic knowledge. According to WAT, abstract concepts are grounded in sensorimotor experience similarly to concrete ones, but are more grounded in linguistic experience than concrete concepts. Therefore, we predict that the different trajectory of decline of concrete and abstract concepts is driven by a different weight of the underlying conceptual components (linguistic, visual, motor etc.). Specifically, we predict a less marked decline of abstract compared to concrete concepts, even if the former are typically acquired later. If, instead, the decline of both kinds of concepts was determined mainly by AoA and frequency effects, abstract concepts should decline faster. This inference, however, needs to be tested empirically.

## Conclusion

Little is known on how our representation of abstract concepts changes with aging. Some tentative evidence shows that abstract concepts deteriorate less than concrete concepts with age, likely because they rely more on language, and vocabulary and semantic knowledge have been shown to be preserved in older. If they deteriorate, compensatory strategies are activated, recruiting neural networks dedicated to phonological processing and, possibly, to visual processing. This evidence is in line with the WAT proposal, according to which abstract concepts activate both perceptual and linguistic information and it is not in line with the hypothesis that AoA and frequency determine the trajectory of decline of abstract concepts. Further empirical research is however necessary to obtain a clearer framework of abstract words mastering in old age.

## Avenues for future research

Based on WAT, we expect that with non-pathological aging, the definitions of abstract concepts will maintain more richness than concrete ones, with reference to social contexts in which they are acquired and used. Considering the different results on the concreteness effect obtained with different tasks, further studies are needed to determine whether and to what extent the time constraints of speed-based tasks will allow for full access to resources such as inner speech characterizing more abstract than concrete concepts. We also propose that an electrophysiological signature of the relative preservation of abstract concepts could be found with EEG, possibly indicated by a modulation of the N400 component of the ERP related to the concreteness effect (West and Holcomb, 2000), and sentence comprehension and predictability (De Long et al., 2012). Importantly, fMRI studies utilizing speeded and non-speeded tasks with concrete and abstract concepts will allow to elucidate whether the hypothesized compensatory mechanisms in the activation of abstract concepts take place.

## Author contributions

Both authors contributed to the conception and writing of this work.

### Conflict of interest statement

The authors declare that the research was conducted in the absence of any commercial or financial relationships that could be construed as a potential conflict of interest.
